# Anticoagulant-Related Nephropathy: A Common, Under-Diagnosed Clinical Entity

**DOI:** 10.7759/cureus.22038

**Published:** 2022-02-08

**Authors:** Agura Afiari, Dimitrios Drekolias, Jason Jacob

**Affiliations:** 1 Department of Nephrology and Hypertension, MedStar Georgetown University Hospital, Washington, USA; 2 Internal Medicine, University of Connecticut Health, Farmington, USA; 3 Internal Medicine, Hartford Hospital, Hartford, USA

**Keywords:** aki, anticoagulant-related nephropathy, medication-induced nephropathy, acute kidney injury, anticoagulation

## Abstract

Anticoagulant-related nephropathy (ARN) is a clinical entity consisting of the constellation of acute kidney injury (AKI) and hematuria in patients receiving anticoagulation therapy. It was formerly known as warfarin-induced nephropathy. The underlying pathogenesis remains yet to be fully clarified. Diagnosis is established by kidney biopsy, which has possibly led to underreporting of the disease. Patients usually present with a supratherapeutic INR. Herein, we present a case of acute kidney injury secondary to anticoagulant-related nephropathy.

## Introduction

The complex interaction and management of comorbidities in patients with chronic kidney disease (CKD) can challenge the balance between renal function preservation and failure. In disease management, the modified dosing of medications can adversely affect the glomerular filtration rate and can increase the risk of adverse reactions. Anticoagulant-related nephropathy (ARN), formerly known as warfarin-induced nephropathy, has been described as acute renal dysfunction with hematuria in patients on anticoagulant medications without any other identifiable cause. When comorbidities are present, renal biopsies become underutilized, which leads to the potential underreporting of ARN in the literature. We present a case of a 57-year-old male who presented with acute kidney injury (AKI) superimposed on CKD secondary to anticoagulant-related nephropathy.

## Case presentation

A 57-year-old male with a history of paroxysmal atrial fibrillation on warfarin, status post permanent pacemaker for sick sinus syndrome, chronic kidney disease stage III, transient ischemic attack, hypertension, type 2 diabetes with diabetic neuropathy, presented with lower extremity edema and fatigue. He reported bilateral lower extremity edema and weight gain of about 20 lbs during the past three weeks. His furosemide was recently increased from 40 mg to 80 mg daily. He denied a change in the volume or color of his urine, gross hematuria, fever, cough, shortness of breath, confusion, rash, pruritus, or dysgeusia.

Lab values on presentation were significant for hemoglobin of 8.7 g/dl, blood urea nitrogen of 109 mg/dL, creatinine of 5.9 mg/dL (compared to 2.61 mg/dL last month), potassium of 4.5 mEq/L, bicarbonate of 23 mEq/L, glucose of 481 mg/dL, albumin 4.0 g/dL, and INR 2.3. The INR was 3.4 a few days prior to the presentation. Pertinent lab work for renal failure, including serum protein electrophoresis (SPEP), anti-nuclear antibodies (ANA), C4, C3, anti-neutrophil cytoplasmic antibodies screen, anti-double strand DNA, HIV, hepatitis B and C, and COVID-19 were negative.

Urinalysis revealed a small amount of protein (30 mg/dl), a large amount of blood, more than 25 red blood cells (RBCs) and four white blood cells per high power field, a random urine microalbumin of 177 mg, and a spot urine protein/creatinine ratio was 0.6 with a urine protein of 33 mg/dL and a urine creatinine of 52 mg/dL.

A chest X-ray did not reveal any acute pulmonary abnormalities. Renal ultrasound revealed increased echogenicity without hydronephrosis or nephrolithiasis. Transthoracic echocardiography showed normal left and right ventricular systolic function with an ejection fraction of 60-65%.

**Figure 1:**
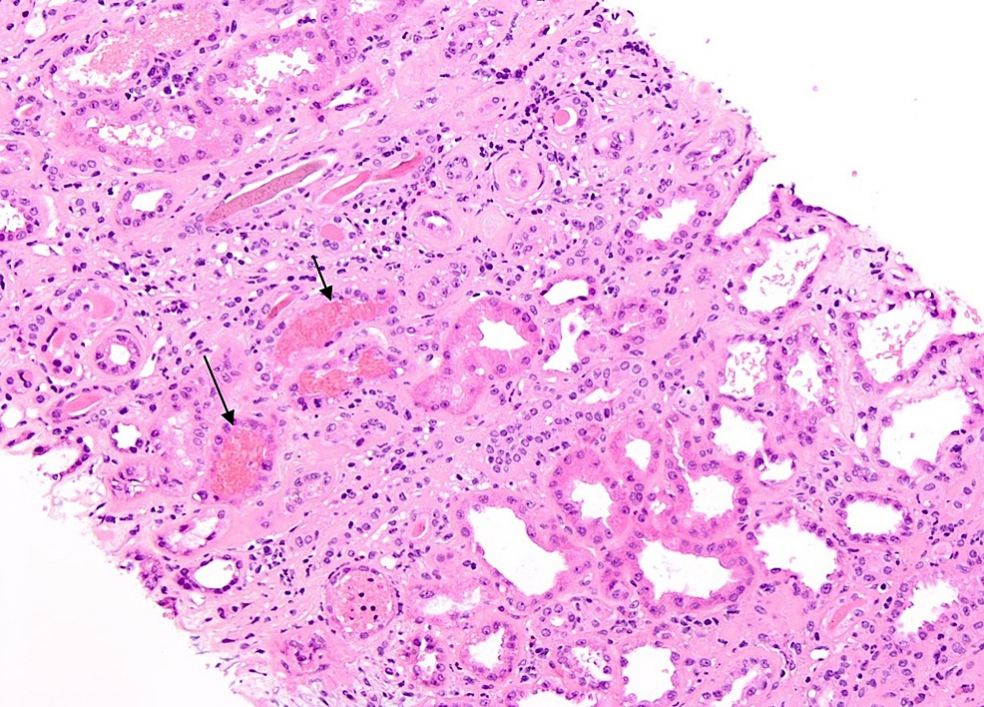
Black arrows indicating intratubular RBCs

The decision was made to obtain a renal biopsy due to the magnitude of symptomatic renal injury within a short period of time and no apparent cause from labs and imaging. Kidney biopsy revealed diffuse, moderate, tubular degenerative and regenerative changes consistent with acute tubular injury with many intratubular red blood cells (Figure 1). Nodular diabetic glomerulosclerosis and interstitial fibrosis were also seen. Minimal mesangial proliferative glomerulonephritis was present and consistent with minimal IgA nephropathy.

Due to persistently elevated uremia with a BUN of 144, the patient was started on hemodialysis, which was continued in the outpatient setting. He was placed on apixaban 2.5 mg twice daily in place of warfarin. His creatinine remained above his prior baseline for at least three months after presentation.

## Discussion

ARN, previously referred to as warfarin nephropathy, is a type of acute kidney injury that has been reported in patients with supratherapeutic INR, and more recently, in patients on newer anticoagulants [1]. The presence of no other obvious cause of acute kidney injury, recent supra-therapeutic INR and hematuria, increased the likelihood of ARN, confirmed by renal biopsy in the above patient.

The true incidence of this entity is difficult to ascertain due to the underreporting and the mixture of other findings seen in kidney biopsies in addition to ARN. The first case of ARN was identified in 2009, despite the use of Warfarin since 1952 [2]. Other factors cited for the underreporting are due to the multifactorial nature of AKI with other medical comorbidities, the higher mortality rate for ARN patients, sicker patients getting tested more frequently, identifying AKI sooner, and caution by the nephrologist to pursue a kidney biopsy while a patient is on anticoagulation [2]. Research has looked at significant risk factors such as supratherapeutic INR and atrial fibrillation as a means of uncovering the true incidence of this entity. In the chronic renal insufficiency cohort (CRIC) study, atrial fibrillation was found to be a strong risk factor for progression to end-stage renal disease (11.8% with CKD to 3.4% without CKD). Brodsky et al. contend that anticoagulation is thought to be a plausible explanation for ARN [2]. One site center experience revealed that 0.5% (41/8636) of kidney biopsies could not be explained by kidney biopsy findings alone if anticoagulation is not considered [3].

Few mechanisms have been proposed to describe the pathophysiology of this underdiagnosed complication of anticoagulant therapy [1]. It is likely not related to prerenal injury, which may occur due to blood loss. Brodsky et al. suggested that patients with chronic kidney disease with already susceptible glomeruli who are exposed to excessive warfarin may have hematuria with RBC casts which lead to tubular obstruction and acute kidney injury [4]. A pattern seen on biopsy will help differentiate bleeding secondary to biopsy procedure (expectedly increased in these patients) from ARN: the absence of active inflammatory lesions, the absence of RBCs at the edges of the biopsy specimen, and the presence of RBCs in the Bowman’s space, as well as RBCs and obstructive RBCs, cast mainly in distal tubules [4]. Abnormalities such as reduced width or dysfunction of the glomerular basement membrane (GBM), which may be seen in diabetes, may also increase the risk of hematuria and RBC cast formation in patients on chronic anticoagulation. This could have played a role in our patient with uncontrolled diabetes and nephrosclerosis seen on biopsy. Thickened GBM may be functionally abnormal and when exposed to anticoagulation may lead to glomerular bleed [5]. This could explain why patients with chronic kidney disease tend to be at a higher risk of ARN. A higher prevalence of warfarin-related nephropathy in CKD patients compared to those without CKD (33% vs 16%) has been documented [6]. Interruption of thrombin-mediated proteinase-activator pathways, which are responsible for the integrity of endothelial cells, may ensue with the use of warfarin, predisposing to glomerular bleeding and leading to ARN [7].

In our case, it is difficult to determine the extent to which the patient’s past medical history contributed to the development of AKI in addition to the ARN. The history of atrial fibrillation, which was present in our case, may have reduced the cardiac output and may have led to a component of prerenal AKI. The patient’s furosemide was increased, placing the patient at risk for overdiuresis and prerenal azotemia. Clinical examination, however, showed volume overload and serum bicarbonate did not appear to show contraction alkalosis. Additionally, the renal dysfunction could be due to the progression of chronic kidney disease secondary to diabetes and hypertension, both present in our case. The protein spot test revealed nephritic and no nephrotic-range proteinuria. Definitive diagnosis is limited without a biopsy in such patients. Obtaining a biopsy could also be a hurdle in the way as medical teams must work closely to determine when there is the least risk of bleeding and the possible need for anticoagulation reversal in these patients who have likely been on chronic anticoagulation. A limitation that could confound the histology results would be the presence of major post-procedural bleeding, which was not present in our case. A CT abdomen done after his renal biopsy showed a small right perinephric hematoma, measuring up to 3.1 cm. No other acute intra-abdominal or pelvic abnormality was seen. No RBCs were reported on the outer edges of the specimen, and the patient had many intratubular RBCs.

The risks, benefits, and alternatives to continuing anticoagulation are to be discussed when there is an adverse reaction to anticoagulation. As an example, patients who are at higher risk for pulmonary embolism may be managed with the placement of an inferior vena cava filter. In patients with a higher CHA2DS2VASc score, the risk of stroke versus the risk of progression of renal disease needs to be carefully weighed. A high risk for stroke was determined given the diagnosis of transient ischemic attack and atrial fibrillation in this case, with a CHA2DS2VASc score of 4. He was started on apixaban at 2.5 mg twice daily with a planned therapeutic dose increase in the event of renal function improvement. However, the literature suggests that patients with ARN will have little to no recovery of kidney function [2].

Although his age and weight were ideal for full-dose therapy, the reduced dose was thought to be a safer option considering the risk of anticoagulant nephropathy with direct oral anticoagulants (DOACs). In a study comparing the risk of AKI between DOACs and warfarin, Shin et al. found a significantly lower risk of acute kidney injury in patients on DOACs compared to those on warfarin [8]. Given the limited data to guide the choice of anticoagulant when considering the risk of ARN, current treatment strategies vary, with close or more frequent monitoring while continuing anticoagulation being the most common approach. Brodsky et al. do recommend a treatment algorithm on what anticoagulant to choose after ARN is treated, recommending a switch to a DOAC if on warfarin, and lowering the dose of the DOAC, if already on DOAC [2].

## Conclusions

Our case highlights the importance of an under-reported diagnosis that may be encountered more frequently than previously thought. The mechanism of renal injury with anticoagulants needs to be fully elucidated. This case highlights the need to have an index of suspicion when a patient presents with acute renal dysfunction and is on anticoagulation with a supratherapeutic INR, especially with warfarin. With continued awareness of this entity, the opportunity also remains to understand the pathophysiology, have a risk prediction model to see which patients are most susceptible to ARN, and create standard treatment guidelines that can be instituted early to restore renal function.
